# Flight performance of pollen starved honey bees and incomplete compensation through ingestion after early life pollen deprivation

**DOI:** 10.3389/fphys.2022.1004150

**Published:** 2022-12-09

**Authors:** Robert Brodschneider, Eslam Omar, Karl Crailsheim

**Affiliations:** ^1^ University of Graz, Institute of Biology, Graz, Austria; ^2^ Plant Protection Department, Faculty of Agriculture, Assiut University, Assiut, Egypt

**Keywords:** flight musculature, protein, consumption, thorax, flight calorimeter, *Apis mellifera*, nutrition

## Abstract

We investigated the effect of adult honey bee pollen nutrition on the flight performance of honey bees. Therefore, caged bees were allowed to perform 30 min of defecation/training flights every second day before flight performance of pollen-fed bees and pollen-deprived bees older than 16 days were compared in a flight mill. We first fed 10 µL of 1 M glucose solution to bees, and after they metabolized this during flight, they were fed 10 µL of 2 M glucose solution for a second flight test. Pollen-deprived bees flew longer and further than pollen-fed bees in both flights. Pollen-fed bees flew faster in the early period at the beginning of flights, whereas pollen-deprived bees were faster in the final phases. Pollen-fed bees were able to raise their maximum flight speed in 2 M glucose solution flights, whereas pollen-constraint bees were not. The two groups did not differ in abdomen fresh weight, but the fresh weight of the head and thorax and dry weight of the head, thorax and abdomen were higher in pollen-fed bees. In a second experiment, we constrained pollen consumption of caged bees during the first 7 days and compared daily consumption of bees from day 8–16 to consumption of bees unrestricted in pollen. We found that pollen-deprived bees perceive the pollen shortage and try to compensate for their needs by consuming significantly more pollen at the later phase of their life than pollen-fed bees of the same age. Still, bees constrained from pollen in the first 7 days did only reach 51.1% of the lifetime consumption of unconstrained bees. This shows that bees can sense the need for essential nutrients from pollen, but their physiological apparatus does not allow them to fully compensate for their early life constraint. Pollen deprivation only in the first 7 days of worker life likewise significantly reduced fresh and dry weights of the body sections (head, thorax, and abdomen) and survival. This underlines the importance of protein consumption in a short critical period early in adult bees’ lives for their development and their performance later in life.

## 1 Introduction

The main natural food sources of honey bees are nectar (or honeydew) and pollen (Brodschneider and Crailsheim, 2010). The two liquid carbohydrate sources provide bees with energy which they need for survival, thermoregulation and flight metabolism. Pollen supplies bees with proteins, lipids, minerals, and vitamins and is needed for brood rearing and adult growth (Schmidt and Buchmann, 1985; Brodschneider and Crailsheim, 2010). Seasonal variations in pollen supply can lead to different nutritive values of the diet for bees, whereby polyfloral diets comprised of the pollen of different flowers are beneficial for honey bee health (di Pasquale et al., 2013; Frias et al., 2016; Omar et al., 2017). De Groot (1953) identified ten essential amino acids that bees need for maximum gain of body mass. Later, Hendriksma et al. (2019) found that nutrition with these essential amino acids is important for developing flight muscles in caged and colony bees. Adult bees, in a colony or cages, consume pollen mainly in the first days after emergence (Crailsheim and Stolberg, 1989; Crailsheim et al., 1992; Omar et al., 2017) and if pollen quality is low, they reach their maximum thorax masses late (Hagedorn and Moeller, 1968).

Fernandez-Winckler and da Cruz-Landim (2008) studied the ultrastructural development of flight muscles in workers of two species of eusocial bees (*Apis mellifera* and *Scaptotrigona postica*). In both species, workers emerge with immature flight muscles and complete their development during the nurse bee stage. The changes during the development of the thorax include synthesis of high numbers of myofibrils, mitochondria and many enzymes for carbohydrate catabolism located in flight muscles (Herold, 1965; Hersch et al., 1978; Suarez, 2000). During flight, honey bees increase their metabolic rate to the top level (Nachtigall et al., 1995; Harrison and Fewell, 2002). Bees fuel the flight almost exclusively with carbohydrates from their honey stomach. Body reserves do not play an important role for flight, as adult bees have comparable low glycogen stores (Gmeinbauer and Crailsheim, 1993; Hrassnigg and Crailsheim, 2005; Hrassnigg et al., 2005). When in need of energy before foraging flights, workers provision themselves with sugars from honey, which they obtain from honey stores or *via* trophallactic contacts (Crailsheim, 1998; Tan et al., 2015).

The consumption of pollen by an individual honey bee worker strongly depends on the age and activities in the colonies. As pointed out before, bees start feeding on beebread soon after they emerge, whereas older bees cease consumption. Young bees are also provided protein-rich jelly from nurse bees by trophallaxis. The high protein turnover of young to middle-aged bees is closely connected to the brood-caring behavior of nurse bees with highly developed hypopharyngeal glands (Dietz, 1969; Crailsheim et al., 1992; Crailsheim, 1998; Amdam et al., 2009). To obtain the proteins from pollen, the midgut of nurse bees has a high proteolytic activity and this enzymatic activity decreases in older foragers, which in turn produce high amounts of carbohydrate digesting enzymes in their hypopharyngeal glands (Moritz and Crailsheim, 1987; Crailsheim et al., 1992; Ohashi et al., 1999). These enzymes accumulate in the midgut and break down even complex sugars. In addition, the microbial community of the midgut and pollen enzymes can contribute to saccharolytic potential (Ricigliano et al., 2017).

As a result of the pollen feeding period early in adult life, protein content and dry weight increase in young bees, and again slightly decrease in older bees (De Groot, 1953). When protein consumption is inadequate, honey bee worker longevity, colony brood area and honey production are reduced. To adapt to environmental pollen shortage, bees in colonies first finish the stored beebread and later exhaust their body reserves to retain brood rearing for a short time before they start cannibalizing larvae (Haydak, 1935; Brodschneider and Crailsheim, 2010). Crailsheim and Stolberg (1989) compared changes in the dry weight of caged and free-flying bees. They found that in free-flying bees dry weight increased until the age of 3 days and remarkably decreased later. In caged bees, dry weight continued to increase until the age of 8 days, probably because caged bees cannot defecate undigested materials. The dry weight of caged bees was highest in bees fed with honey and beebread compared to several other *in vitro* diets. The dry weight of bees deprived of protein in cages for 3 days and then transferred to colonies did not differ much from hive controls, suggesting some ability to compensate for early pollen starvation.Early developmental nutrition profoundly influences phenotypic trajectories and affects adult physiology, behavior, longevity, and fitness in many animal species (Buchanan et al., 2022). For bees, Crailsheim and Stolberg (1989) investigated the effect of delaying the protein nutrition on the levels of proteolytic activities and the size of the hypopharyngeal glands. Still, nothing is known about the perception, attempts and abilities of bees compensating for pollen starvation during this critical period early in adult life by feeding more pollen later in life. Therefore we studied, if bees are 1) sensing a nutritional deficiency experienced in early adult life (which could optionally, but not necessarily, result in an increased consumption later in life) and 2) if such an experimentally induced delayed consumption is successful in satisfying the bees’ lifetime protein intake. As the early starvation and possible compensation later in life might be too late for showing an effect on the development of hypopharyngeal glands needed at young age, we measured thorax weight as an indicator for nutrient assimilation and flight muscles (Hendriksma et al., 2019; Ricigliano and Simone-Finstrom, 2020; Ricigliano et al., 2022). Regarding macronutrient ratios, social insects do have astounding abilities to anticipate and react towards certain intake goals (Simpson and Raubenheimer, 2009; Vaudo et al., 2016). Bees may even be able to select a certain forage source that complements another available diet which is deficient in a particular amino acid (Hendriksma and Shafir, 2016). However, the ability of bees of adjusting delayed food intake after pollen starvation during early life has not been studied before. We designed an experiment to measure dynamics of pollen consumption of bees unrestricted in pollen and constrained to delayed pollen consumption. The daily pollen ingestion of bees in this experiment informs us if bees with a delayed consumption attempt to compensate for early pollen starvation. If they do, lifetime consumption informs us, if they can fully compensate this deficit.

Larval diet quality has been shown to affect adult worker bees’ flight performance (Brodschneider et al., 2009) and flight onset in drones (Metz and Tarpy, 2022). Here we investigate if adult protein nutrition as well affects flight muscle development and flight ability of the honey bee. We, therefore developed a test in which we could control adult bee protein nutrition in cages but allow bees to defecate and develop their flight apparatus in ‘training flights’ outside the cage. Our second aim was to study if caged honey bees, deprived of pollen during the first days of life, would attempt to compensate for their needs in a later life phase, and if they can satisfy their protein needs then.

## 2 Materials and methods

### 2.1 Honey bees and pollen diet

Honey bee workers were obtained by incubating sealed brood combs of different, unrelated *Apis mellifera carnica* colonies at 34.5°C under standard conditions (Williams et al., 2013; Omar et al., 2017). Each comb was placed in a separate comb cage. Newly emerged bees (0–24 h old) from these brood combs were mixed and randomly introduced into adult bee cages. Two different types of cages were used in the two different experiments, see below for specifications. Pollen loads for preparing pollen diet were collected using front-mounted plastic pollen traps (Anel, Greece) on several days from colonies at the same apiary and frozen at -20°C. A homogeneous mixture of this pollen of several colonies and undefined polyfloral botanical origin was made and was kneaded with 10% (w/w) water until a homogeneous pollen dough was formed (Williams et al., 2013). The dough was further stored at -20°C and thawed on the day of use. The pollen diet was presented in one-half of a cylindrical 10 ml plastic tube. Standard carbohydrate feeding (50% w/v sucrose solution) was provided *ad libitum* in 1.5 ml punctured Eppendorf vials and renewed daily.

### 2.2 Experiment 1: Effect of adult nutrition on flight performance

For this experiment we used wooden cages (15 × 15 × 5.5 cm) covered with a wooden board from one side, which could be opened to remove bees and change diets. The other side was covered with a grid that allowed air ventilation. Each cage was provided with a piece of wax for bees to cluster on. Four cages were used in this experiment, each cage containing 60 newly emerged honey bees. The four cages were divided into two groups with two replicates each and kept in an incubator at 34.5°C. The two cages from the first group received sugar solution as described above and pollen diet *ad libitum* during the first 16 days of the experiment (pollen-fed bees). The pollen diet was renewed every day. The two cages from the second group received sugar solution only (pollen-deprived bees).

#### 2.2.1 Flight training

**FIGURE 1:**
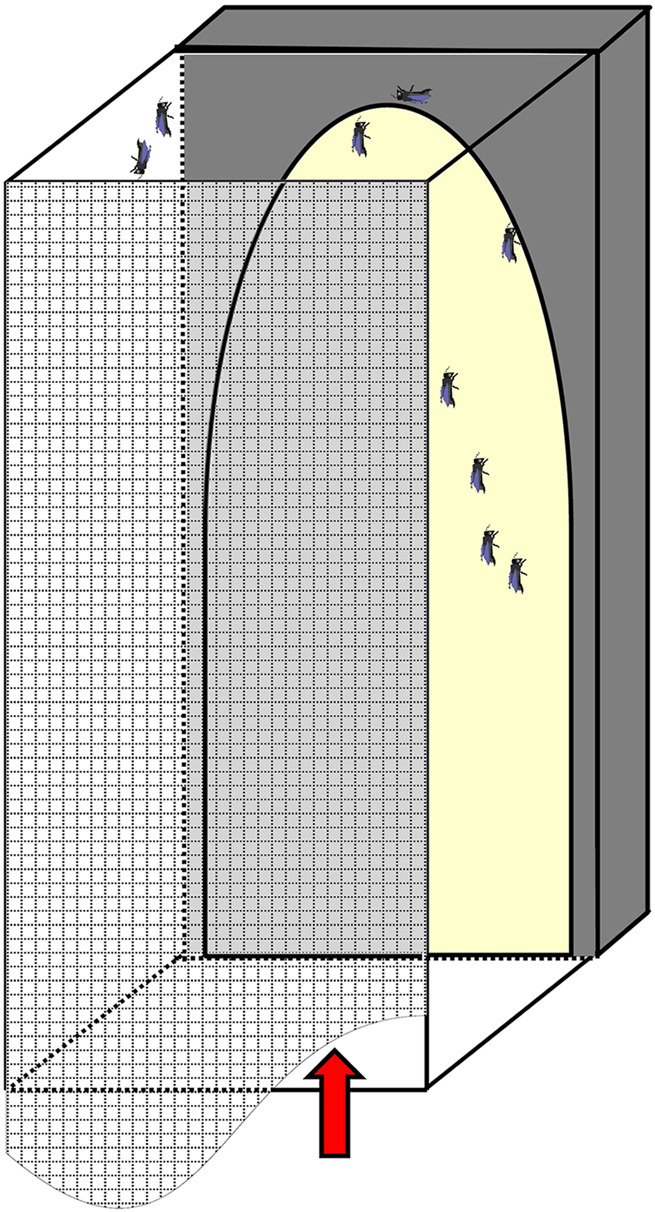
Glass box used for flight training. The box was covered with mosquito net (meshed) and light trap (yellow) on the back. Arrow indicates where bees are introduced.

From day three on and every 2 days thereafter, we allowed bees from both groups separately to fly free and defecate for 30 min in a 30 × 30 × 60 cm glass box (Figure 1). On one side of the glass box a light trap attracted bees to fly to the light and from the other side it could be closed by a net to prevent the flying bees from escaping. After the flight training of the first group was finished, bees were collected by a modified hand-held vacuum cleaner, by hand or with forceps and put back in their belonging cages, before the next group of bees was released for flight training. This way, the two groups of bees could not be mixed up, and no marking of bees was necessary.

#### 2.2.2 Flight experiments

We tested the flight performance of caged bees following the protocols in Brodschneider et al. (2009) and Scheiner et al. (2013). We started flight experiments when the caged bees reached the age of 16 days and continued daily until day 29. Alternatingly, one bee from the group of pollen-deprived bees followed by a pollen-fed bee was taken for flight experiments. Each bee was attached by a small tube on the thorax to the 14.5 cm long arm of a flight mill (roundabout), so one rotation covered 91.06 cm. The attached bee was repeatedly stimulated to begin the flight by removing a small ball of paper that the bee held with her legs. Workers that did not start a continuous flight within 20 min were noted down for calculating the rate of (un-)successful flyers and discarded.

After an emptying flight which forces the bee to spend most of its sugar reserves from the gut and the hemolymph, the bee was fed 10 μL of 1 M glucose solution with a micro-pipette and given a resting time of exactly 5 minutes. Before and after feeding, bees were weighed on an analytical balance to the nearest 0.1 mg, to confirm complete ingestion of the glucose solution. Then the bee was stimulated to fly and empty all its sugar reserves (first flight, 1 M). The number of rotations, duration per rotation flown by the bee in the flight mill was recorded by a computer, and the overall flight time was additionally clocked by hand, so that only the active flight period was considered in further calculations. After the bee ran out of energy (defined as not being able to move the arm of the flight mill any more) the same bee received 10 μL of 2 M glucose solution (second flight, 2 M) and the flight procedure was repeated as above.

Because ambient temperature affects flight metabolic rate and speed (Hrassnigg and Crailsheim, 1999), the temperature in the flight mill was measured in real time and automatically logged for every rotation of the flight mill. Temperature was manually maintained at around 26°C using a 40W light bulb in a lamp above the flight mill. This lamp could be placed closer to increase, or further away from the flight mill to decrease the temperature.

Flight distance, average speed, maximum speed per minute and mean metabolic power were calculated from the number of rounds logged by the flight mill and the radius of the flight mill, the active flight time, the amount of glucose in feedings and the food energy in glucose (15.7kJ per g) (Gmeinbauer and Crailsheim, 1993; Brodschneider et al., 2009). From the flight mill log data, the maximum speed per round was extracted for each flight. We calculated average speed for different periods of the flights, which were cut into three periods (10 min each) for 1 M flights, or four periods (10 min each, except the last one, which was 15 min) for 2 M flights.

After the two flights, each bee was dissected into the head, thorax (including legs and wings), and abdomen, and each part was weighed to the nearest 0.1 mg. The sections were dried for 7 days in an incubator at 70°C and dry weight was measured to the nearest 0.1 mg (Human et al., 2013).

### 2.3 Experiment 2: Compensation of early pollen deprivation

The experimental cages in this experiment consisted of clear 0.3 L plastic cups with two holes, one in the base of the cup and the other on the side of the cup. The cages were supplied with a wax comb. The cage was closed from below by a grid that allowed air to pass through (Evans et al., 2009). After we placed bees into the cage, we covered the hole in the base with a tape. The other hole was closed with a 1.5 ml Eppendorf vial in which three small holes had been punctured from which the bees could drink sucrose solution. The pollen diet was presented in one-half of a cylindrical 10 ml plastic tube, as in the previous experiment. All bees in cages were kept in the dark in an incubator at 34.5°C for 16 days.

Six hundred newly emerged honey bees younger than 24 h from the same origin as explained above were randomly distributed into twelve cages, 50 bees per cage. The twelve cages were divided into two groups of six replicates each. The first group received pollen diet from day one until day 16 (‘full access’). The second group did not receive pollen in the first 7 days, but from day eight until day 16 (‘constraint access’). Sucrose solution was applied *ad libitum* and renewed daily. Dead bees were counted daily and removed from the cages. Every day we renewed the pollen diet. To measure the amount consumed, the difference between the weight of diet applied and the weight after bees consumed was measured on an analytical balance to the nearest 0.1 mg. To calculate food consumption per bee, dead bees were assumed not to have consumed any food since the prior food was changed. The period during which bees had access to the protein diet was measured to the minute but was recalculated to reflect 24 h consumption (Williams et al., 2013; Omar et al., 2017). We calculated cumulative consumption per bee for the group always allowed feeding on pollen (‘full access, day 1–16’), and additionally for the second phase of this experiment only (‘full access, day 8–16’, ignoring the consumption on the first 7 days). This allowed comparing the normal consumption of bees older than 8 days (‘full access, day 8–16’) to bees given access to pollen only on days 8–16 (‘constraint access, day 8–16’). Cumulative consumption per bee on day 16 represents the lifetime consumption of bees in this experiment and informs us if bees with a delayed consumption attempt to and are able to compensate early life pollen starvation.

Dead bees were removed and counted daily to analyze survival. After 16 days, the fresh and dry weight of body sections of ten randomly chosen bees from each cage were measured the same as in the first experiment.

### 2.4 Statistics

We compared the rate of successful flights of pollen-fed and pollen-deprived bees with two-sided Pearson’s chi-square test. Age of bees used for flight experiments and all flight parameters (duration, distance, average and maximum speed, ambient temperature) followed a normal distribution (one-sample Kolmogorov-Smirnov test, *p* > 0.07) with unequal variances in some variables (Levene statistic, *p* = 0.00–0.95). We therefore compared groups using two-tailed Student’s *t*-test assuming or not assuming equal variances, where applicable. First and second flights of same bees were compared with two-tailed paired-samples Student’s *t*-tests.

Fresh and dry weight of body sections followed a normal distribution (one-sample Kolmogorov-Smirnov test, *p* > 0.1) with unequal variances in some variables (Levene statistic, *p* = 0.02–0.73). We therefore compared groups using two-tailed Student’s *t*-tests assuming or not assuming equal variances, where applicable.

Consumption data of experiment 2 (compensation of early pollen deprivation) followed a normal distribution (one-sample Kolmogorov-Smirnov test, *p* > 0.7) with homogeneity of variances (Levene statistic, *p* > 0.3) and was therefore compared using one-way ANOVA with Bonferroni correction for multiple comparisons. Daily pollen consumptions per bee on each of day eight to 16 followed a normal distribution (one-sample Kolmogorov-Smirnov test, *p* > 0.3) with unequal variances in some variables (Levene statistic, *p* = 0.04–0.82) and were compared using two-tailed Student’s *t*-test assuming or not assuming equal variances, where applicable. We compared survival of bees with full and constraint consumption with Mantel-Cox log-rank tests. All statistical analyses were made in SPSS Statistics version 21 (IBM).

## 3 Results

### 3.1 Experiment 1: Effect of adult nutrition on flight performance

**FIGURE 2:**
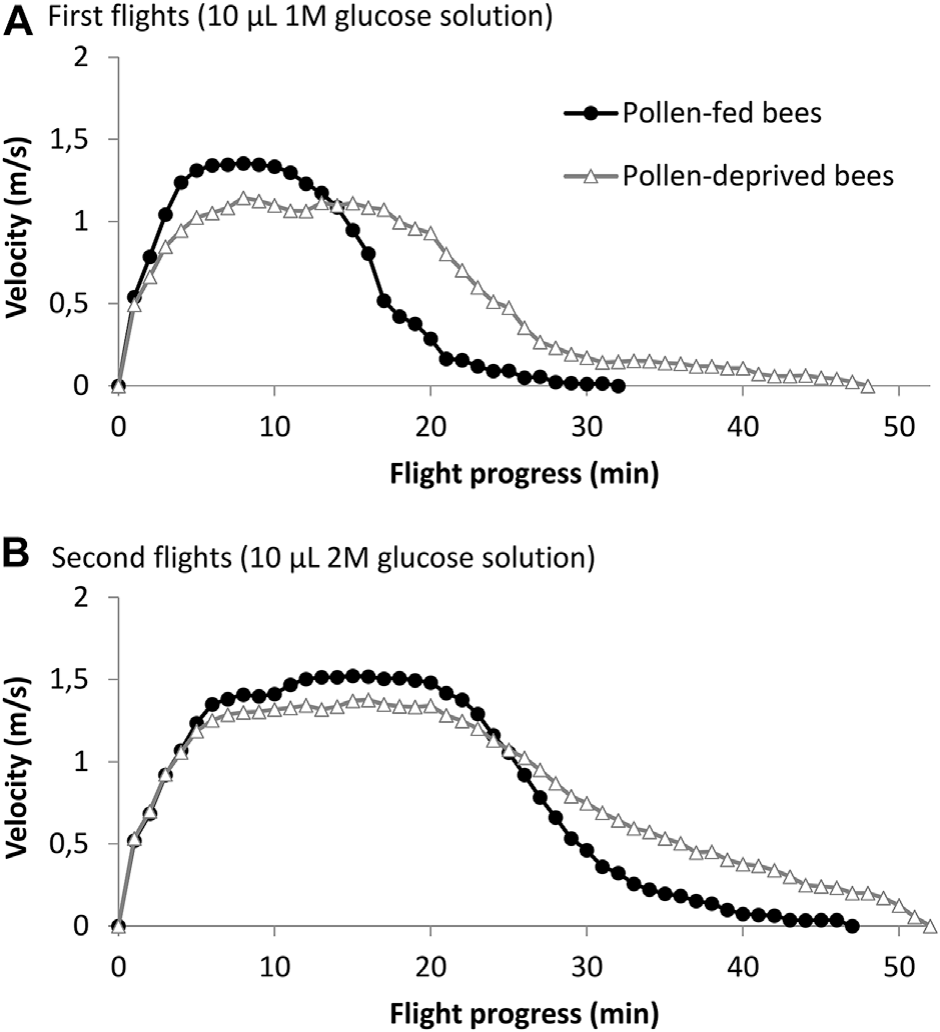
Mean flight speed of pollen-fed and pollen-deprived bees. **(A)** After being fed 10 μL of 1 M glucose solution (*n* = 23 bees per group) and **(B)** 10 μL of 2 M glucose solution (*n* = 23 pollen-fed bees and 18 pollen-deprived bees).

Of all tested bees, 83.6% were capable to fly in the flight mill. 88.5% (23 out of 26) of bees that received a pollen diet successfully flew in the flight mill, compared to 79.3% (23 out of 29) of bees that were pollen-deprived. Flight success rates did not differ between the two groups (Χ^2^ = 0.36, *n* = 55, *p* > 0.05). Five in the first flight successful bees of the pollen-deprived group failed to fly in the 2 M flight. The age of bees used for flight experiments, just as the temperature during flight experiments, was successfully controlled not to differ between the two groups, (*p* > 0.1, Student’s *t*-test, Table 1). Flight data (flight speed for each minute of the flight, averaged for all individuals) for both groups and the two different glucose feedings are shown in Figure 2. Pollen-deprived bees flew longer and further than pollen-fed bees in both flights (*p* < 0.05, Student’s *t*-test, Table 1). Both groups did not differ in their average speed for the entire period of flight, but the pollen-fed bees flew significantly faster in the first 10-minutes-period of acceleration at the beginning of the 1 M flights (*p* < 0.001), but not in 2 M flights (*p* = 0.084, Student’s *t*-test, Table 1). Pollen-deprived bees flew significantly faster in the rest of the flight periods of both flights (second and third periods in the 1 M flight and second, third and fourth periods in the 2 M flight, Table 1).

**TABLE 1:** Flight parameters of pollen-fed bees and pollen-deprived bees in two feeding regimes (10 μL of 1 M glucose solution, 10 μL of 2 M glucose solution).

Feeding regime		Pollen-fed bees	Pollen-deprived bees	*p*
10 μL 1 M glucose	N	23	23	
	Age (d)	22.8 ± 4.1	22.7 ± 4.3	0.917
	Duration (sec.)	1,048.3 ± 205.1	1,308.2 ± 374.2	0.006
	Distance (m)	1,063.0 ± 283.6	1,280.4 ± 411.8	0.044
	Average speed (m/s)	1.0 ± 0.3	1.0 ± 0.2	0.545
	Average speed minute 1 to 10 (m/s)	1.16 ± 0.46	0.95 ± 0.43	<0.001
	Average speed minute 11 to 20 (m/s)	1.05 ± 0.4	1.13 ± 0.38	0.029
	Average speed minute 21 to 31 (m/s)	0.59 ± 0.2	0.96 ± 0.35	<0.001
	Maximum speed per minute (m/s)	1.5 ± 0.4	1.5 ± 0.2	0.838
	Maximum speed per round (m/s)	1.6 ± 0.4	1.7 ± 0.5	0.415
	Ambient temperature (°C)	26.3 ± 0.7	25.9 ± 1.0	0.196
10 μL 2 M glucose	N	23	18	
	Age (d)	22.8 ± 4.1	23 ± 4.7	0.875
	Duration (Sec.)	1708.6 ± 299.2	2,622.5 ± 1,131.4	0.003
	Distance (m)	2032.6 ± 517.0	2,942.2 ± 1,165.2	0.005
	Average speed (m/s)	1.2 ± 0.3	1.2 ± 0.2	0.571
	Average speed minute 1 to 10 (m/s)	1.14 ± 0.52	1.06 ± 0.43	0.084
	Average speed minute 11 to 20 (m/s)	1.5 ± 0.44	1.22 ± 0.34	<0.001
	Average speed minute 21 to 30 (m/s)	1.16 ± 0.44	1.31 ± 0.33	<0.001
	Average speed minute 31 to 46 (m/s)	0.84 ± 0.27	1.24 ± 0.37	<0.001
	Maximum speed per minute (m/s)	1.6 ± 0.4	1.6 ± 0.3	0.553
	Maximum speed per round (m/s)	1.7 ± 0.5	1.6 ± 0.3	0.402
	Ambient temperature (°C)	26.7 ± 0.6	26.6 ± 0.8	0.847

Means and standard deviations, sample sizes, and *p*-values (two-tailed Student’s *t*-tests) are given. For paired comparisons between 1 M and 2 M flights, see results.

For both, pollen-fed and pollen-deprived bees, time, distance, and average speed were higher in 2 M flights than in 1 M flights (*p* < 0.05, paired-samples Student’s *t*-tests). Maximum speed per minute and per round of pollen-fed bees was higher in 2 M flights than in 1 M flights (*p* < 0.05, paired-samples Student’s *t*-tests) whereas we found no differences in maximum speed per minute or per round between flights with 1 M and 2 M in pollen-deprived bees (*p* > 0.05, paired-samples Student’s *t*-tests). Average speeds of the first and second period of flights were also higher in second flights with 2 M glucose feeding compared to flights with 1 M glucose feeding (*p* < 0.05, paired-samples Student’s *t*-tests), except in 1 M flights of pollen-deprived bees, where *p* = 0.070.

Knowing the flight duration and the amount of spent (=fed) glucose, we calculated the mean (± standard deviation) metabolic power of honey bee flight (Gmeinbauer and Crailsheim, 1993; Nachtigall et al., 1995; Brodschneider et al., 2009). This was 28.0 ± 5.9 mW (*n* = 23) for pollen-fed bees and 23.6 ± 7.6 mW (*n* = 23) for pollen-deprived bees in 1 M flights. For 2 M glucose solution flights, the mean metabolic power was 34.0 ± 5.6 mW (*n* = 23) for pollen-fed bees and 27.8 ± 19.2 mW (*n* = 18) for pollen-deprived bees.

**FIGURE 3:**
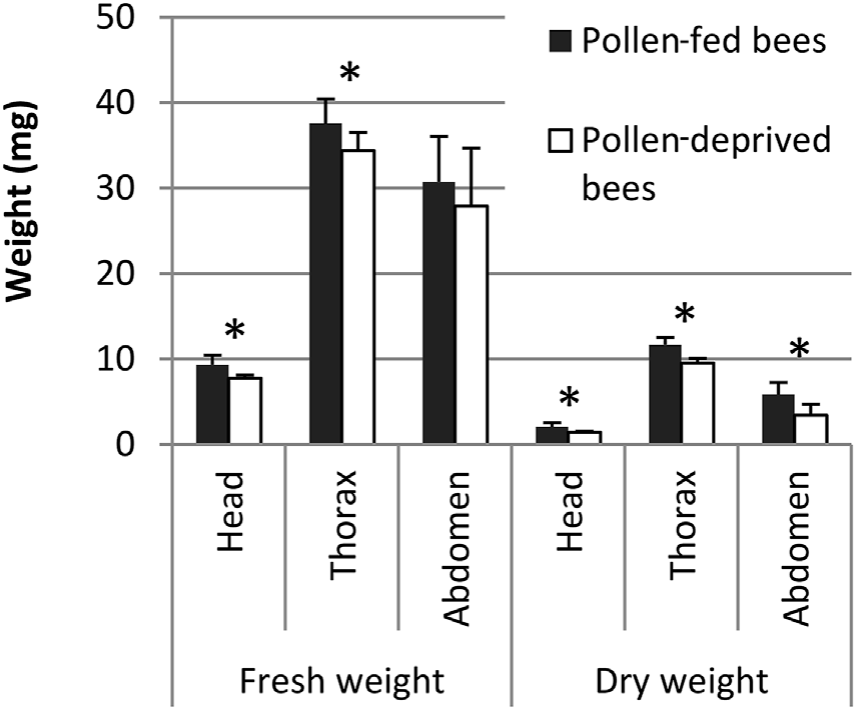
Fresh and dry weight of the head, thorax, and abdomen of pollen-fed bees and pollen-deprived bees from flight experiments. Means and standard deviations are shown: *n* = 24–26 bees. * indicates *p* < 0.05, Student’s *t*-test.

Pollen-fed and pollen-deprived bees did not differ in abdomen fresh weight (*p* > 0.05, Student’s *t*-test, Figure 3), but the fresh weight of head and thorax were significantly higher in pollen-fed bees (*p* < 0.05, Student’s *t*-test). Dry weights of the head, thorax and abdomen were significantly higher in pollen-fed than in pollen-deprived bees (*n* = 24–26, the non-flying bees were also included in weight measurements, *p* < 0.05, Student’s *t*-test, Figure 3).

### 3.2 Experiment 2: Compensation of early pollen deprivation

**FIGURE 4:**
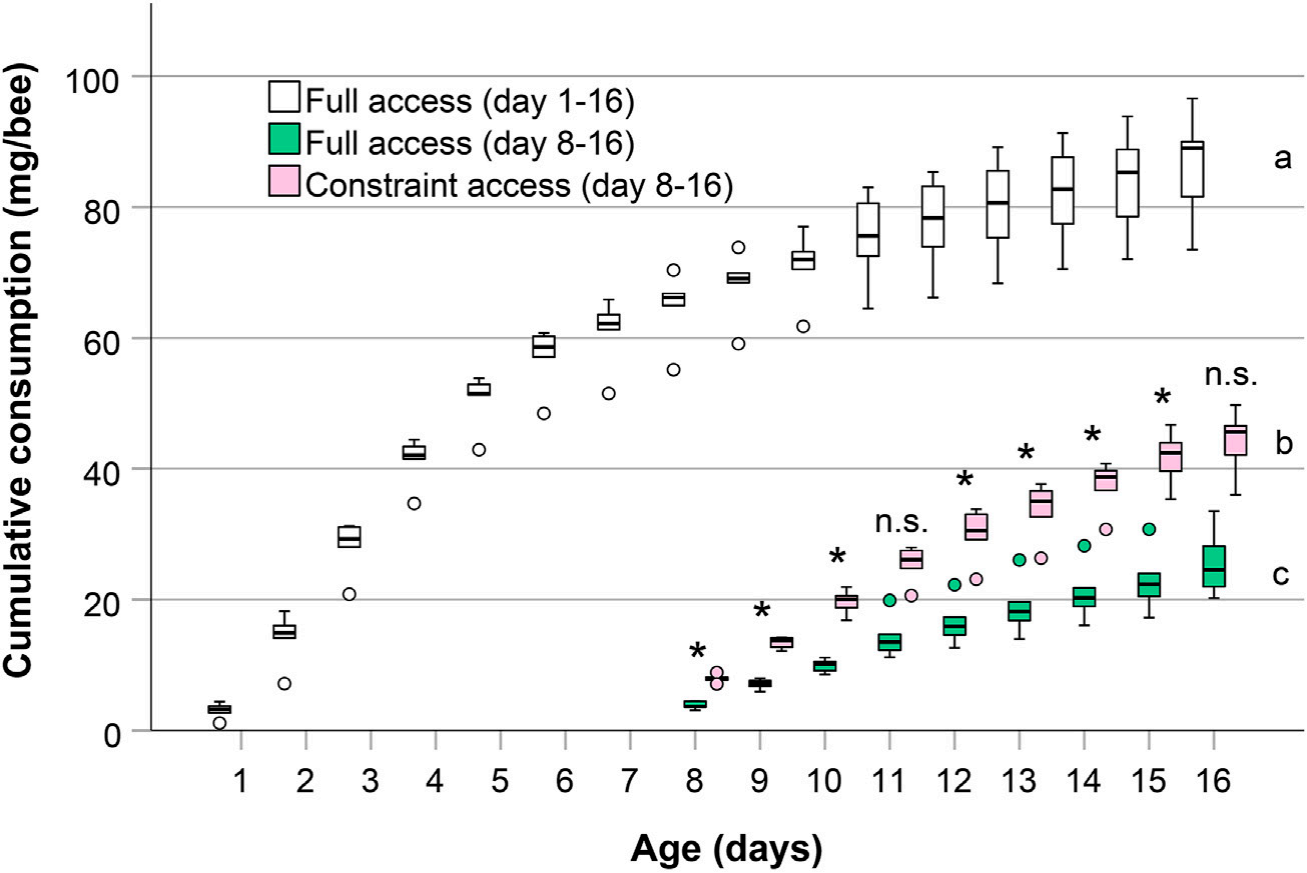
Cumulative pollen consumption of bees with unrestricted access and bees constrained of pollen during the first 7 days. For comparison between groups at the later stage, consumption of unrestricted bees on days 8–16 is shown seperately. Box and whisker plots (minimum, lower quartile, median, upper quartile, maximum and outliers) for cumulative consumption of pollen (mg/bee) by caged honey bees from day 1 until day 16 and from day 8 until day 16, respectively (*n* = 6 cages of bees for each group). Cumulative lifetime consumption at age 16 days was significantly different (a, b, c: different letters indicate *p* < 0.001, one-way ANOVA with Bonferroni multiple comparisons post-hoc test). * indicates differences in daily consumption after day 8 between bees with constraint consumption and full consumption (*p* < 0.05, Student’s *t*-test). n.s. = not significant.

We found significant differences between all cumulative lifetime consumptions. An individual worker bee in a cage with full pollen supply cumulatively consumed a mean of 86.6 mg in the first 16 days of her life, the late life consumption in the period from day 8–16 was 25.5 mg (see boxplots for day 16 in Figure 4). Bees prevented from pollen consumption in early life consumed a mean of 44.3 mg pollen in total in the period from day 8 to day 16. The cumulative lifetime consumption of bees with full pollen supply (‘full access, day 1–16’), bees allowed feeding on pollen only after day eight (‘constraint access, day 8–16’) and the amount that unrestricted bees consumed in the second half of the experiment (‘full access, days 8–16’) were all significantly different from each other (*n* = 6 each, *p* < 0.001, one-way ANOVA with Bonferroni multiple comparisons post-hoc test, Figure 4). Also, daily pollen intakes after day 8 were significantly higher in bees not allowed feeding pollen for the first 7 days compared to unrestricted bees (*p* < 0.05, Student’s *t*-test, Figure 4), except on days 11 and 16 (*p* > 0.05).

**FIGURE 5:**
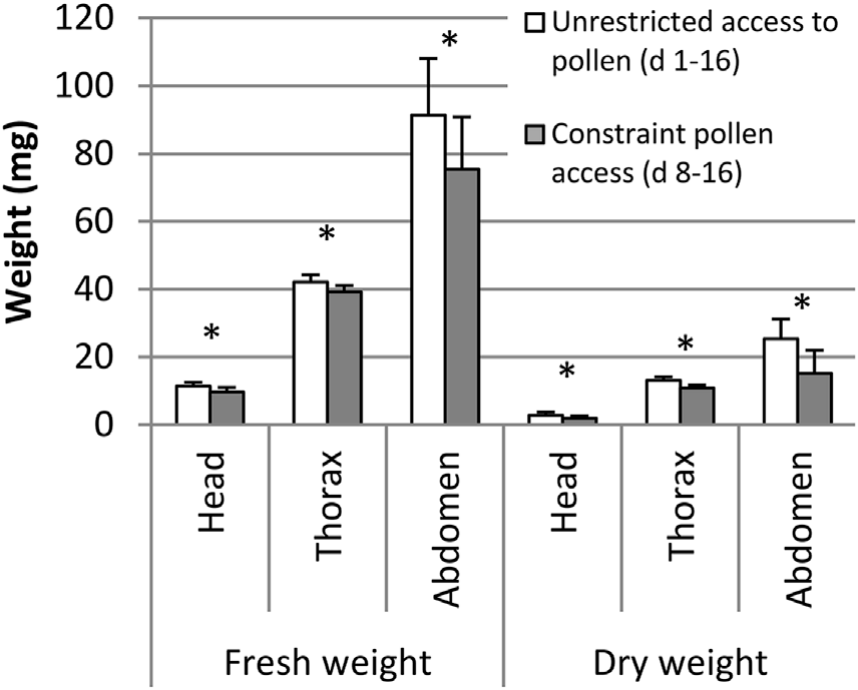
Fresh and dry weight of head, thorax, and abdomen of bees with unrestricted access to pollen (day 1–16) and constraint pollen access (day 8–16). Means and standard deviations are shown: *n* = 60 (10 bees out of 6 replicated cages). * indicates *p* < 0.05, Student’s *t*-test.

Mortality during the 16 days experiment was higher (17.7%, *n* = 300) in bees deprived of pollen in the first 7 days compared to bees allowed full pollen supply (7.3%, *n* = 300, *p* < 0.05, Mantel-Cox log-rank test). At the end of the experiment, 16-day-old adult bees deprived of pollen for the first 7 days had lower fresh and dry weights of the heads, thorax or abdomen compared to bees unrestricted in pollen access (*n* = 60, *p* < 0.05, Student’s *t*-test, Figure 5).

## 4 Discussion

Larval protein provisioning is important for the development of the flight apparatus of honey bees (Brodschneider et al., 2009). Still, the development of flight muscles in the honey bee thorax is ongoing after adult emergence (Herold, 1965; Hersch et al., 1978; Harrison, 1986; Roberts and Elekonich, 2005; Fernandez-Winckler and da Cruz-Landim, 2008). Young bees feed on beebread, and this source of proteins, lipids and other nutrients is attributed to better health status (Alaux et al., 2010; DeGrandi-Hofmann et al., 2010), internal gland development (Hrassnigg and Crailsheim, 1998) and increasing thorax and dry weight (Hagedorn and Moeller, 1968; Crailsheim and Stolberg, 1989). So far, no experiment connected adult protein provisioning and physiological performance of the flight muscle in honey bees. We closed this gap by investigating the importance of adult pollen nutrition for honey bee flight in a flight mill bioassay. We had to develop a new method of keeping bees *in vitro* for flight experiments, as previous trials of flight experiments with caged honey bees failed (R. Brodschneider, unpublished data). Because healthy bees do neither defecate in their nest, nor in a cage (see Pavlović et al., 2022), the ventriculus (midgut) and rectum of caged bees are densely filled with undigested pollen, water, and feces. The pressure of the well-stocked intestine on the tracheal system (including collapsible air sacs) hinders optimal oxygen delivery and hence flight. To overcome this, we developed the flight training box used in this experiment (Figure 1). From the age of 3 days on, bees were every second day allowed to complete 30 min periods of flight training and defecation flights. The regular defecation in training flights (visible by spots of feces on the glass walls of the flight box) reduced the fresh weight of abdomens of caged bees for about 50 mg (compare weights of caged bees in Figure 3 with weights of caged bees from experiment 2 not allowed defecation flights in Figure 5, though those bees were of different ages). The flight trainings consequently resulted in a high rate of successfully flying bees. Another validation for emptying the bees’ intestine with the defecation flights is that the fresh abdominal weight between pollen-fed and pollen-deprived bees did not differ (Figure 3), though this was after the extensive flight experiments in which bees also often defecated.

Adult bees deprived of pollen significantly differ from pollen-fed bees in several flight characteristics: pollen-deprived bees flew with lower metabolic power longer and further than pollen-fed bees, they showed lower flight speed at the beginning of the flights, (Table 1), and they could not raise maximum flight speed in 2 M glucose flights compared to 1 M glucose flights. Bees fly longer and further when energy-richer 2 M glucose is fed compared to 1 M glucose solution, but they also increase speed (von Frisch and Lindauer, 1955; Gmeinbauer and Crailsheim, 1993; Brodschneider et al., 2009). The flight curves (Figure 2) show that the first minutes of flights are characterized by powerful and fast flight, and bees afterwards steadily reduce speed until carbohydrate reserves in the intestine and hemolymph are spent (Gmeinbauer and Crailsheim, 1993; Hrassnigg et al., 2005). We interpret the longer (and thus further) flights of pollen deprived bees in their lowered ability for high energy turnover in this period at the beginning of flights. Pollen-deprived bees are probably impaired in their post-emergence flight muscle maturation or digestive and other physiological processes (see Ricigliano et al., 2017) which probably limit performing full force. Still, all bees received the same amount of sugar fuel, so pollen-deprived bees spend less fuel per time of flight and hence flew longer and further in both experimental flights. The longer flight times with the same energetic expenditure are also reflected in lower metabolic power (Nachtigall et al., 1995) in pollen-deprived bees, very similar to experiments where larval nutrition was manipulated (Brodschneider et al., 2009).

The flight capacity of honey bees has been proven to be affected by different biotic and abiotic factors. Some or even all the factors discussed below could synergistically act together and drastically impair the flight capacity of bees, which subsequently reduces collected forage and colony fitness. In this study we demonstrated the negative impact of pollen malnutrition during adult bee life, whereas the effect of larval nutrition was shown in Brodschneider et al. (2009). We can speculate that the worst case for flight capacity development would be that bees experience deficient nutrition during both, larval and early adult development. Pesticides can further impair honey bee flight by reducing respiration or mitochondrial activity (Hatjina et al., 2013; Nicodemo et al., 2014; Tosi et al., 2017; Coulon et al., 2020). In bumble bees, Syromyatnikov et al. (2017) reported that fungicides used in greenhouses inhibit mitochondria in the flight muscle. The neonicotinoid pesticide imidacloprid reduced range and period of bumble bee flights (Kenna et al., 2019). High, but not field-realistic concentrations of a mito-toxic fungicide did impair honey bee flight capacity in a flight chamber (Glass et al., 2021). These authors by the way also reported a reduced thorax mass of bees reared under field-realistic fungicide concentrations. Next to the abiotic factors, several honey bee pests may affect flight physiology. Blanken et al. (2015) showed that forager bees fly shorter distances when parasitized with the mite *Varroa destructor* and exposed to imidacloprid, a neonicotinoid insecticide, but flight speed remained unaffected. Another parasitic mite living in the honey bees’ tracheas, *Acarapis woodi*, reduces the safety margin for tracheal oxygen delivery during flight in hypoxic air (Harrison et al., 2001). Dosselli et al. (2016) reported that infection with the microspiridium *Nosema apis* affects honey bee workers flight activity. In contrast to this, Wells et al. (2016) did not confirm this for bees naturally infected with *Nosema ceranae*, but demonstrated that infection with deformed wing virus reduced flight time and distance.

Some flight characteristics were significantly different between the two investigated groups, but our results also show that even adult bees completely deprived of pollen nutrition can fly. Larval feeding, pupal development and compromised post-emergence development of the thorax obviously suffice for flight, even without adult provision of pollen derived nutrients. This underlines the importance of flight musculature for honey bees. We assume a relatively strong need for adult maturation of the important flight muscle compared to other organs. In bees, the allocation tradeoff of nutritional resources in distress, and possible competitions of different organs or body parts, largely remain to be explored (Nijhout and Emlen, 1998; Tigreros and Davidowitz, 2019; Metz and Tarpy, 2022). We need to emphasize that in our tethered flight experiment bees of both groups were quasi forced to fly, especially at the latter periods of flights. Slight drawbacks in flight of honey bees in free-flying environments could be more dramatic, as bees may fail in returning to the colony after unsuccessful attempts (Harrison et al., 2001). Pollen substitutes optimized for the bees’ needs could partly support their thorax development when there is insufficient or only nutritionally deficient pollen available in the environment (Pavlović et al., 2022; Ricigliano et al., 2022). In our experiment we established a very drastic all-or-nothing situation regarding pollen availability of the two experimental groups. Such long periods of complete pollen starvation may be rare in a colony with its beebread stores, but we can speculate if a sensitive short phase for pollen feeding exists in a young worker bee’s life, which we investigated in the second experiment.

In this feeding experiment we tested if bees perceive a complete protein deficit experienced during the first days after emergence and compensate for, or at least try to compensate for. Such a compensation could be manifested in an increased consumption at a later stage, to levels higher than measured in bees of the same age, but never restricted in pollen. A certain flexibility in age-related behavior or gland development is common in honey bees (Crailsheim und Stolberg, 1989; Robinson, 1992; Hrassnigg and Crailsheim, 1998; Schmickl and Crailsheim, 2004). Indeed, bees prevented from feeding on pollen in the first 7 days of their life subsequently fed more pollen on almost every day at ages 8–16 days, compared to bees that always had full access to pollen (Figure 4). This evidences that the bees perceive the pollen deficit and increase consumption to compensate for the early life deprivation. Nonetheless, the cumulative lifetime pollen intake of bees deprived of pollen the first 7 days resulted at the end of the experiment on day 16 in only about half (51.1%) of the amount of pollen ingested compared to bees unrestricted in pollen. If the experiment would have lasted a few days longer, the lifetime consumption would have only increased marginally, as can be seen by the flattening of the cumulative consumption curve (Figure 4). As expected, caged bees having full access to pollen consumed most of the pollen at the first 5 days of their life (Dietz, 1969; Crailsheim et al., 1992; Omar et al., 2017). The reduced lifetime pollen consumption of constraint bees suggests that older bees’ physiology does not allow them to catch up with the protein consumption missed during early life. Reasons for this could be the transition in ability of older bees to digest pollen diets, the atrophy of hypopharyngeal glands or the absence of protein in the bees’ early diet which could influence digestion or metabolism (Moritz and Crailsheim, 1987; Crailsheim and Stolberg, 1989; Ricigliano et al., 2017). Because of the critical phase for adult pollen nutrition identified in our study, some age cohorts in colonies may already suffer from shorter periods of pollen dearth than previously thought. For these distressed bees, even a good pollen forage after a severe shortage is not a solid remedy of the malnourishment (Requier et al., 2017).

In both experiments, full and early-life restrictions to protein-free diets resulted in reduced body weight of worker bees. This was particularly the case for the fresh and dry weight of the thorax, site of the flight muscles. In the second experiment we constraint bees of pollen only for the first 7 days of their life, when bees usually consume pollen (Dietz, 1969; Moritz and Crailsheim, 1987; Crailsheim et al., 1992). Deprivation at this early age is sufficient to significantly reduce thorax weight. A delayed availability of protein food for bees older than 7 days did not allow full thorax weight compensation of the deficits experienced during early life, whereas starvation during only the first 3 days of life could be compensated later (Crailsheim and Stolberg, 1989). Aside from the thorax, also head weight is reduced, which is an indication for impaired hypopharyngeal gland development (Hrassnigg and Crailsheim, 1998). Bees feeding on pollen later in life than usual experience early adult life stress (malnutrition in a sensitive feeding period) which affects body weight and probably life history.

Accordingly, we found that early adult life pollen deprivation for 7 days is enough to stunt honey bee survival *in vitro*, whereas significant impacts on longevity so far have mostly been established for life-long pollen deprivation (Omar et al., 2017; Khedidji et al., 2022) or when bees were offered only a lowered share (40%) of the amount of oilseed rape pollen usually consumed (di Pasquale et al., 2016). Consequences of early life stress in honey bees have so far been mostly investigated by manipulating parasite or nutrition levels during the developmental stages (Scofield and Mattila, 2015; Rueppel et al., 2017). Amdam et al. (2009) on the contrary studied brood-rearing levels of young adult bees and found these to be linked to life trajectories. They found that no brood-rearing activity of young bees is associated with high vitellogenin protein levels, a later onset of foraging and longer life expectancy. In our experiment bees experienced nutritional stress in their early adult life which caused lower body weight and longevity. Such bees may be an interesting new study subject for scientific research of early life deprivation or as an intermediate group between fully fed and unfed bees.

Our findings underline the importance of nutrients from pollen for the development of adult honey bees. We provide evidence for the need of adult pollen nutrition in terms of flight muscle mass, and maximal flight force. We further detected a sensitive phase of pollen feeding in the first 7 days of adult life of honey bees. Bees deprived of pollen during this critical period for protein consumption can perceive this shortcoming and try to compensate it by increasing their daily pollen consumption later in life. Unfortunately, their age-related physiological constitution does not allow them to consume enough pollen to reach a full lifetime amount of protein. The deficiencies acquired during this early adult-life deprivation cannot be fully compensated as we showed for body mass and longevity and possibly affect honey bee life trajectories and health.

## Data Availability

The raw data supporting the conclusion of this article will be made available by the authors, without undue reservation.

## References

[B1] Alaux C. Ducloz F. Crauser D. Le Conte Y. (2010). Diet effects on honeybee immunocompetence. Biol. Lett. 6 (4), 562–565. 10.1098/rsbl.2009.0986 20089536 PMC2936196

[B2] Amdam G. V. Rueppell O. Fondrk M. K. Page R. E. Nelson C. M. (2009). The nurse’s load: Early-life exposure to brood-rearing affects behavior and lifespan in honey bees (*Apis mellifera*). Exp. Gerontol. 44 (6-7), 467–471. 10.1016/j.exger.2009.02.013 19264121 PMC3798062

[B3] Blanken L. J. van Langevelde F. van Dooremalen C. (2015). Interaction between *Varroa destructor* and imidacloprid reduces flight capacity of honeybees. Proc. Biol. Sci. 282 (1820), 20151738. 10.1098/rspb.2015.1738 26631559 PMC4685773

[B4] Brodschneider R. Crailsheim K. (2010). Nutrition and health in honey bees. Apidologie 41, 278–294. 10.1051/apido/2010012

[B5] Brodschneider R. Riessberger-Gallé U. Crailsheim K. (2009). Flight performance of artificially reared honeybees (*Apis mellifera*). Apidologie 40, 441–449. 10.1051/apido/2009006

[B6] Buchanan K. L. Meillère A. Jessop T. S. (2022). “Early life nutrition and the programming of the phenotype,” in Development strategies and biodiversity. Fascinating life sciences. Editors Costantini D. Marasco V. (Cham: Springer). 10.1007/978-3-030-90131-8_6

[B7] Coulon M. Dalmon A. Di Prisco G. Prado A. Arban F. Dubois E. (2020). Interactions between thiamethoxam and deformed wing virus can drastically impair flight behavior of honey bees. Front. Microbiol. 11, 766. 10.3389/fmicb.2020.00766 32425910 PMC7203464

[B8] Crailsheim K. Schneider L. H. W. Hrassnigg N. Bühlmann G. Brosch U. Gmeinbauer R. (1992). Pollen consumption and utilization in worker honeybees (*Apis mellifera carnica*): Dependence on individual age and function. J. Insect Physiology 38 (6), 409–419. 10.1016/0022-1910(92)90117-v

[B9] Crailsheim K. Stolberg E. (1989). Influence of diet, age and colony condition upon intestinal proteolytic activity and size of the hypopharyngeal glands in the honeybee (*Apis mellifera* L). J. Insect Physiology 35, 595–602. 10.1016/0022-1910(89)90121-2

[B10] Crailsheim K. (1998). Trophallactic interactions in the adult honeybee (*Apis mellifera* L). Apidologie 29, 97–112. 10.1051/apido:19980106

[B11] De Groot A. P. (1953). Protein and amino acid requirements of the honey bee. Physiol. Comp. Oecol. 3, 1–90.

[B12] DeGrandi-Hoffman G. Chen Y. Huang E. Huang M. H. (2010). The effect of diet on protein concentration, hypopharyngeal gland development and virus load in worker honey bees (*Apis mellifera* L). J. Insect Physiol. 56, 1184–1191. 10.1016/j.jinsphys.2010.03.017 20346950

[B13] Di Pasquale G. Alaux C. Le Conte Y. Odoux J. F. Pioz M. Vaissière B. E. (2016). Variations in the availability of pollen resources affect honey bee health. PloS one 11 (9), e0162818. 10.1371/journal.pone.0162818 27631605 PMC5025243

[B14] Di Pasquale G. Salignon M. Le Conte Y. Belzunces L. P. Decourtye A. Kretzschmar A. (2013). Influence of pollen nutrition on honey bee health: Do pollen quality and diversity matter? PloS one 8 (8), e72016. 10.1371/journal.pone.0072016 23940803 PMC3733843

[B15] Dietz A. (1969). Initiation of pollen consumption and pollen movement through the alimentary canal of newly emerged honey bees. Ann. Entomological Soc. Am. 62 (1), 43–46. 10.1093/aesa/62.1.43

[B16] Dosselli R. Grassl J. Carson A. Simmons L. W. Baer B. (2016). Flight behaviour of honey bee (*Apis mellifera*) workers is altered by initial infections of the fungal parasite *Nosema apis* . Sci. Rep. 6, 36649. 10.1038/srep36649 27827404 PMC5101476

[B17] Evans J. D. Chen Y. P. Di Prisco G. Pettis J. Williams V. (2009). Bee cups: Single-use cages for honey bee experiments. J. Apic. Res. 48 (4), 300–302. 10.1080/00218839.2009.11101548

[B18] Fernandez-Winckler F. da Cruz-Landim C. (2008). A morphological view of the relationship between indirect flight muscle maturation and the flying needs of two species of advanced eusocial bees. Micron 39 (8), 1235–1242. 10.1016/j.micron.2008.04.004 18672375

[B19] Frias B. E. D. Barbosa C. D. Lourenço A. P. (2016). Pollen nutrition in honey bees (*Apis mellifera*): Impact on adult health. Apidologie 47 (1), 15–25. 10.1007/s13592-015-0373-y

[B20] Glass J. R. Fisher A. II Fewell J. H. DeGrandi-Hoffman G. Ozturk C. Harrison J. F. (2021). Consumption of field-realistic doses of a widely used mito-toxic fungicide reduces thorax mass but does not negatively impact flight capacities of the honey bee (*Apis mellifera*). Environ. Pollut. 274, 116533. 10.1016/j.envpol.2021.116533 33529906

[B21] Gmeinbauer R. Crailsheim K. (1993). Glucose utilization during flight of honeybee (*Apis mellifera*) workers, drones and queens. J. Insect Physiology 39, 959–967. 10.1016/0022-1910(93)90005-c

[B22] Hagedorn H. H. Moeller F. E. (1968). Effect of the age of pollen used in pollen supplements on their nutritive value for the honeybee. I. Effect on thoracic weight, development of hypopharyngeal glands, and brood rearing. J. Apic. Res. 7, 89–95. 10.1080/00218839.1968.11100195

[B23] Harrison J. (1986). Caste-specific changes in honeybee flight capacity. Physiol. Zool. 59 (2), 175–187. 10.1086/physzool.59.2.30156031

[B24] Harrison J. F. Camazine S. Marden J. H. Kirkton S. D. Rozo A. Yang X. (2001). Mite not make it home: Tracheal mites reduce the safety margin for oxygen delivery of flying honeybees. J. Exp. Biol. 204 (4), 805–814. 10.1242/jeb.204.4.805 11171363

[B25] Harrison J. F. Fewell J. H. (2002). Environmental and genetic influences on flight metabolic rate in the honey bee, *Apis mellifera* . Comp. Biochem. Physiol. A Mol. Integr. Physiol. 133 (2), 323–333. 10.1016/s1095-6433(02)00163-0 12208303

[B26] Hatjina F. Papaefthimiou C. Charistos L. Dogaroglu T. Bouga M. Emmanouil C. (2013). Sublethal doses of imidacloprid decreased size of hypopharyngeal glands and respiratory rhythm of honeybees *in vivo* . Apidologie 44 (4), 467–480. 10.1007/s13592-013-0199-4

[B27] Haydak M. H. (1935). Brood rearing by honeybees confined to a pure carbohydrate diet. J. Econ. Entomology 28, 657–660. 10.1093/jee/28.4.657

[B28] Hendriksma H. P. Pachow C. D. Nieh J. C. (2019). Effects of essential amino acid supplementation to promote honey bee gland and muscle development in cages and colonies. J. Insect Physiol. 117, 103906. 10.1016/j.jinsphys.2019.103906 31254521

[B29] Hendriksma H. P. Shafir S. (2016). Honey bee foragers balance colony nutritional deficiencies. Behav. Ecol. Sociobiol. 70 (4), 509–517. 10.1007/s00265-016-2067-5

[B30] Herold R. C. (1965). Development and ultrastructural changes of sarcosomes during honey bee flight muscle development. Dev. Biol. 12 (2), 269–286. 10.1016/0012-1606(65)90031-x 5884146

[B31] Hersch M. I. Crewe R. M. Hepburn H. R. Thompson P. R. Savage N. (1978). Sequential development of glycolytic competence in the muscles of worker honeybees. Comp. Biochem. Physiology Part B Comp. Biochem. 61 (3), 427–431. 10.1016/0305-0491(78)90149-9

[B32] Hrassnigg N. Brodschneider R. Fleischmann P. H. Crailsheim K. (2005). Unlike nectar foragers, honeybee drones (*Apis mellifera*) are not able to utilize starch as fuel for flight. Apidologie 36, 547–557. 10.1051/apido:2005042

[B33] Hrassnigg N. Crailsheim K. (1998). Adaptation of hypopharyngeal gland development to the brood status of honeybee (*Apis mellifera* L.) colonies. J. Insect Physiol. 44, 929–939. 10.1016/s0022-1910(98)00058-4 12770429

[B34] Hrassnigg N. Crailsheim K. (2005). Differences in drone and worker physiology in honeybees (*Apis mellifera* L). Apidologie 36, 255–277. 10.1051/apido:2005015

[B35] Hrassnigg N. Crailsheim K. (1999). Stoffwechselraten und metabolische Leistung von Honigbienen im Fesselflug in Abhängigkeit von Temperatur und Luftwiderstand (Hymenoptera: Apidae). Entomol. Gen. 24, 23–30. 10.1127/entom.gen/24/1999/23

[B36] Human H. Brodschneider R. Dietemann V. Dively G. Ellis J. D. Forsgren E. (2013). Miscellaneous standard methods for *Apis mellifera* research. J. Apic. Res. 52 (4), 1–18. 10.3896/ibra.1.52.4.09

[B37] Kenna D. Cooley H. Pretelli I. Ramos Rodrigues A. Gill S. D. Gill R. J. (2019). Pesticide exposure affects flight dynamics and reduces flight endurance in bumblebees. Ecol. Evol. 9 (10), 5637–5650. 10.1002/ece3.5143 31160987 PMC6540668

[B38] Khedidji H. Abderrahmani K. Oulebsir-Mohandkaci H. Ladjali-Mohammedi K. Mohammedi A. (2022). Effects of pollen deprivation in groups of tellian (*Apis mellifera intermissa*) and saharan (*Apis mellifera sahariensis*) honey bees under controlled conditions. Insects 13 (8), 727. 10.3390/insects13080727 36005352 PMC9409310

[B39] Metz B. N. Tarpy D. R. (2022). Variation in the reproductive quality of honey bee males affects their age of flight attempt. PeerJ 10, e13859. 10.7717/peerj.13859 35935251 PMC9354755

[B40] Moritz B. Crailsheim K. (1987). Physiology of protein digestion in the midgut of the honeybee (*Apis mellifera* L). J. Insect Physiology 33, 923–931. 10.1016/0022-1910(87)90004-7

[B41] Nachtigall W. Hanauer-Thieser U. Mörz M. (1995). Flight of the honey bee VII: Metabolic power versus flight speed relation. J. Comp. Physiol. B 165 (6), 484–489. 10.1007/bf00261303

[B42] Nicodemo D. Maioli M. A. Medeiros H. C. Guelfi M. Balieira K. V. De Jong D. (2014). Fipronil and imidacloprid reduce honeybee mitochondrial activity. Environ. Toxicol. Chem. 33 (9), 2070–2075. 10.1002/etc.2655 25131894

[B43] Nijhout H. F. Emlen D. J. (1998). Competition among body parts in the development and evolution of insect morphology. Proc. Natl. Acad. Sci. U. S. A. 95 (7), 3685–3689. 10.1073/pnas.95.7.3685 9520426 PMC19896

[B44] Ohashi K. Natori S. Kubo T. (1999). Expression of amylase and glucose oxidase in the hypopharyngeal gland with an age-dependent role change of the worker honey bee (*Apis mellifera* L). Eur. J. Biochem. 265 (1), 127–133. 10.1046/j.1432-1327.1999.00696.x 10491166

[B45] Omar E. Abd-Ella A. A. Khodairy M. M. Moosbeckhofer R. Crailsheim K. Brodschneider R. (2017). Influence of different pollen diets on the development of hypopharyngeal glands and size of acid gland sacs in caged honey bees (*Apis mellifera*). Apidologie 48 (4), 425–436. 10.1007/s13592-016-0487-x

[B46] Pavlović R. Dojnov B. Šokarda Slavić M. Pavlović M. Slomo K. Ristović M. (2022). In pursuit of the ultimate pollen substitute (insect larvae) for honey bee (*Apis mellifera*) feed. J. Apic. Res. 2022, 1–10. 10.1080/00218839.2022.2080950

[B47] Requier F. Odoux J. F. Henry M. Bretagnolle V. (2017). The carry-over effects of pollen shortage decrease the survival of honeybee colonies in farmlands. J. Appl. Ecol. 54 (4), 1161–1170. 10.1111/1365-2664.12836

[B48] Ricigliano V. A. Fitz W. Copeland D. C. Mott B. M. Maes P. Floyd A. S. (2017). The impact of pollen consumption on honey bee (*Apis mellifera*) digestive physiology and carbohydrate metabolism. Arch. Insect Biochem. Physiol. 96 (2), e21406. 10.1002/arch.21406 28833462

[B49] Ricigliano V. A. Simone-Finstrom M. (2020). Nutritional and prebiotic efficacy of the microalga *Arthrospira platensis* (spirulina) in honey bees. Apidologie 51 (5), 898–910. 10.1007/s13592-020-00770-5

[B50] Ricigliano V. A. Williams S. T. Oliver R. (2022). Effects of different artificial diets on commercial honey bee colony performance, health biomarkers, and gut microbiota. BMC Vet. Res. 18 (1), 52–14. 10.1186/s12917-022-03151-5 35062935 PMC8780706

[B51] Roberts S. P. Elekonich M. M. (2005). Muscle biochemistry and the ontogeny of flight capacity during behavioral development in the honey bee, *Apis mellifera* . J. Exp. Biol. 208 (22), 4193–4198. 10.1242/jeb.01862 16272241

[B52] Robinson G. E. (1992). Regulation of division of labor in insect societies. Annu. Rev. Entomol. 37 (1), 637–665. 10.1146/annurev.en.37.010192.003225 1539941

[B53] Rueppell O. Yousefi B. Collazo J. Smith D. (2017). Early life stress affects mortality rate more than social behavior, gene expression or oxidative damage in honey bee workers. Exp. Gerontol. 90, 19–25. 10.1016/j.exger.2017.01.015 28122251 PMC5346452

[B54] Scheiner R. Abramson C. I. Brodschneider R. Crailsheim K. Farina W. M. Fuchs S. (2013). Standard methods for behavioural studies of *Apis mellifera* . J. Apic. Res. 52 (4), 1–58. 10.3896/ibra.1.52.4.04

[B55] Schmickl T. Crailsheim K. (2004). Inner nest homeostasis in a changing environment with special emphasis on honey bee brood nursing and pollen supply. Apidologie 35, 249–263. 10.1051/apido:2004019

[B56] Schmidt J. O. Buchmann S. L. (1985). Pollen digestion and nitrogen utilization by *Apis mellifera* L. (Hymenoptera: Apidae). Comp. Biochem. Physiology Part A Physiology 82 (3), 499–503. 10.1016/0300-9629(85)90423-2

[B57] Scofield H. N. Mattila H. R. (2015). Honey bee workers that are pollen stressed as larvae become poor foragers and waggle dancers as adults. PLoS One 10, e0121731. 10.1371/journal.pone.0121731 25853902 PMC4390236

[B58] Simpson S. J. Raubenheimer D. (2009). Macronutrient balance and lifespan. Aging (Albany NY) 1 (10), 875–880. 10.18632/aging.100098 20157561 PMC2815731

[B59] Suarez R. K. (2000). Energy metabolism during insect flight: Biochemical design and physiological performance. Physiol. Biochem. Zool. 73 (6), 765–771. 10.1086/318112 11121349

[B60] Syromyatnikov M. Y. Kokina A. V. Lopatin A. V. Starkov A. A. Popov V. N. (2017). Evaluation of the toxicity of fungicides to flight muscle mitochondria of bumblebee (*Bombus terrestris* L). Pestic. Biochem. Physiol. 135, 41–46. 10.1016/j.pestbp.2016.06.007 28043329

[B61] Tan K. Latty T. Dong S. Liu X. Wang C. Oldroyd B. P. (2015). Individual honey bee (*Apis cerana*) foragers adjust their fuel load to match variability in forage reward. Sci. Rep. 5, 16418. 10.1038/srep16418 26549746 PMC4637910

[B62] Tigreros N. Davidowitz G. (2019). Flight-fecundity tradeoffs in wing-monomorphic insects. Adv. insect physiology 56, 1–41. 10.1016/bs.aiip.2019.02.001

[B63] Tosi S. Burgio G. Nieh J. C. (2017). A common neonicotinoid pesticide, thiamethoxam, impairs honey bee flight ability. Sci. Rep. 7, 1201. 10.1038/s41598-017-01361-8 28446783 PMC5430654

[B64] Vaudo A. D. Patch H. M. Mortensen D. A. Tooker J. F. Grozinger C. M. (2016). Macronutrient ratios in pollen shape bumble bee (*Bombus impatiens*) foraging strategies and floral preferences. Proc. Natl. Acad. Sci. U. S. A. 113 (28), E4035–E4042. 10.1073/pnas.1606101113 27357683 PMC4948365

[B65] von Frisch K. Lindauer M. (1955). Über die Fluggeschwindigkeit der Bienen und über ihre Richtungsweisung bei Seitenwind. Naturwissenschaften 42, 377–385. 10.1007/bf00640847

[B66] Wells T. Wolf S. Nicholls E. Groll H. Lim K. S. Clark S. J. (2016). Flight performance of actively foraging honey bees is reduced by a common pathogen. Environ. Microbiol. Rep. 8 (5), 728–737. 10.1111/1758-2229.12434 27337097 PMC5091639

[B67] Williams G. R. Alaux C. Costa C. Csaki T. Doublet V. Eisenhardt D. (2013). Standard methods for maintaining adult *Apis mellifera* in cages under *in vitro* laboratory conditions. J. Apic. Res. 52 (1), 1–36. 10.3896/ibra.1.52.1.04

